# Association between cumulative psychosocial adversity in the family and ADHD and autism: a family-based cohort study

**DOI:** 10.1038/s41398-023-02571-7

**Published:** 2023-08-14

**Authors:** Aleksandra Kanina, Henrik Larsson, Arvid Sjölander, Agnieszka Butwicka, Mark J. Taylor, Miriam I. Martini, Paul Lichtenstein, Frida E. Lundberg, Brian M. D’ Onofrio, Mina A. Rosenqvist

**Affiliations:** 1 Department of Medical Epidemiology & Biostatistics, Karolinska Institutet, Stockholm, Sweden; 2 School of medical sciences, Örebro University, Örebro, Sweden; 3 Institute of Clinical Medicine, University of Oslo, Oslo, Norway; 4 Division of Mental Health Services, Akershus University Hospital, Lørenskog, Norway; 5 Department of Biostatistics and Translational Medicine, Medical University of Lodz, Lodz, Poland; 6 Department of Psychological and Brain Sciences, Indiana University, Bloomington, IN USA

**Keywords:** ADHD, Autism spectrum disorders

## Abstract

Cumulative exposure to psychosocial adversity at an early age has been shown to be a risk factor for attention-deficit hyperactivity disorder (ADHD) and autism that often co-occur. However, it is not clear if this association reflects a causal effect or familial confounding. We aimed to assess whether cumulative psychosocial adversity in the family increases the risk for ADHD and autism in offspring while accounting for unmeasured familial confounding. We used a population-based cohort of 1,877,901 individuals born in Sweden between 1990 and 2009. Participants were followed from the age of 3 until 2013, with a median follow up time of 13.8 years. We created a cumulative index based on 7 psychosocial adversity factors. We used Cox regression to estimate the hazard ratios (HRs) relating neurodevelopmental conditions to cumulative psychosocial adversity. To address familial confounding, the analyses were repeated in groups of relatives of different kinship: siblings and half-siblings and cousins. A dose-response relationship was observed between cumulative exposure to psychosocial adversity and ADHD at a general population level (covariate adjusted HRs (aHRs) with 95% confidence intervals ranged from 1.55 [one adversity; 1.53–1.58] to 2.65 [ ≥ 4 adversities; 1.98–3.54]). No clear dose-response relation was seen for autism (aHRs ranged from 1.04 [.59–1.84] to 1.37 [1.30–1.45]). HRs of ADHD and autism decreased with increasing level of kinship in the analysis of relatives. Cumulative exposure to psychosocial adversity was associated with both ADHD and autism in the general population, these associations were partly explained by unmeasured familial confounding between relatives. This highlights the need for using family-based designs in studies of psychosocial adversity and ADHD and autism.

## Introduction

Attention-deficit hyperactivity disorder (ADHD) and autism are common neurodevelopmental conditions, which first manifest in early childhood and often co-occur [1]. Both ADHD and autism are highly heritable with heritability estimates around 70% for ADHD [2] and up to 90% for autism [3]. Up to 70% of autistic individuals are diagnosed with ADHD and around 13% of individuals with ADHD receive a diagnosis of autism [4–6]. Furthermore, there are many previously reported risk factors associated with ADHD and autism related to the child themself (e.g. prematurity, low birth weight), pregnancy and delivery complications, and to parental factors (age, stress during pregnancy and social adversity) [2, 7]. Psychosocial adversity, reflecting stress that arises due to adverse life events that an individual experiences, has also been associated with neurodevelopmental conditions [8]. However, few studies have investigated the effect of cumulative psychosocial adversity in the family on neurodevelopmental outcomes and the role of unmeasured confounding.

A scale for psychosocial adversity created by Rutter et al. consists of several factors: severe marital discord, low socioeconomic status, large family size, paternal criminality, maternal mental condition, and foster care placement [9]. Based on Rutter’s index, Biederman et al. found that aggregation of those factors increases the risk for ADHD [10]. Similarly, several observational studies and a meta-analysis have shown that the risk for ADHD increases with the number of psychosocial adversities [11, 12]. In addition, a Danish population-based cohort study that assessed the indicators of adversity in the first year of life, reported similar results describing a statistically significant dose-response effect of psychosocial adversity (such as marital discord, large family size, paternal criminality, maternal mental condition and placement in out-of-home care) on risk for ADHD among male and female individuals [8]. Exposure to psychosocial adversity in early childhood is also associated with increased risk of autism, however studies on cumulative psychosocial adversity are scarce [13, 14].

When attempting to establish causal associations, residual confounding through both genetic and environmental factors must be considered. One major limitation of previous studies of psychosocial adversity and neurodevelopmental conditions is that most were unable to adjust for unmeasured familial (i.e., genetic and shared environmental factors) confounding. This is critical because psychosocial adversity [15], as well as diagnosis of ADHD and autism, are influenced by genetic factors. Therefore, unless unmeasured familial confounding is considered, there is a remaining possibility for a spurious association caused by unmeasured residual confounding. An English and Romanian adoptee study reported on outcomes of Romanian children born in the 1980s who stayed in an orphanage with poor conditions for 43 months before adoption while partly accounting for unmeasured confounding [16]. Those who were exposed to the severe adversity in the institution for longer than 6 months were more likely to be diagnosed with both ADHD and autism. This highlights the effect of extreme forms of psychosocial adversity (e.g., “poor hygiene, insufficient food, little personalized care, and social and cognitive understimulation” [17]) during early childhood [16]. However, further research is needed using genetically sensitive study designs to clarify if less severe forms of cumulative psychosocial adversity have similar effects on ADHD and autism after accounting for unmeasured confounding. Family-based designs is another example of a genetically informative design, where differentially exposed groups of relatives are compared [18]. For example, sibling, half-sibling and cousin comparisons, an increasingly recognized approach to adjust for unmeasured familial confounding [19], could be used by comparing the risk of neurodevelopmental conditions within family members that are differentially exposed to psychosocial adversity.

By choosing the first year of life as an exposure period, we are minimizing the risk of reverse causation. Families of children with neurodevelopmental conditions experience higher levels of stress and are more likely to experience psychosocial adversity [20]. This is a major limitation of the previous research, since most studies concentrate on the preschool and school age, there is a possibility for remaining reverse causation [21].

Moreover, ADHD and autism often co-occur; the rate of ADHD in individuals with autism varies from 25.7% to 70% [5, 22], and autism diagnosis is prevalent in up to 13–66% of people with ADHD [5, 23]. Findings show that this could be due to partly shared etiology of ADHD and autism [24], but the extent to which environmental risk factors have specific or general effects on different neurodevelopmental conditions remains largely unknown [25]. More specifically, it is currently unclear if psychosocial adversity at an early age is more directly associated with ADHD or autism.

The aim of this study was to investigate the association between cumulative psychosocial adversity in the family during the first year of life and neurodevelopmental conditions in offspring, using family-based study designs to control for unmeasured confounding, and to assess if psychosocial adversity is specifically associated with ADHD or autism.

## Materials and methods

### Data sources and study population

Data for this study were obtained by linking different Swedish population-based registers using personal identity numbers [26]: the Total Population Register contains information about all individuals living in Sweden since 1968 [27]; the Medical Birth Register includes information on all deliveries in Sweden since 1973 [27]; the Swedish National Patient Register (NPR) covers more than 99% of somatic and psychiatric in-patient care records since 1987 and out-patient records from specialist health care since 2001 [28]; the Prescribed Drug Register (PDR) covers all filled drug prescriptions from 2005 onwards [29]; the Longitudinal Integrated Database for Health Insurance and Labor Market Studies (LISA) was established in 1990 and contains information on socioeconomic factors such as education, income, employment and occupation [30]; information on convictions comes from the National Crime Register (Swedish National Council for Crime Prevention); and the Multi-Generation Register links different generations through the personal identity number [27].

Using these registers, we identified all individuals born in Sweden between 1 January 1990 (due to information availability from LISA) and 31 December 2009 (*n* = 2,042,257) as well as their parents and followed them until 31 December 2013. We excluded individuals who were stillborn or had major congenital malformations, twins, everyone who died or migrated during the first year of life, and individuals with missing information on their own or their parents’ personal identity number. After exclusion, the analytical sample consisted of 1,877,901 individuals. Among these, we identified subgroups of full siblings (1,271,883 individuals from 568,058 families) for the sibling comparison, half-siblings (304,881 individuals from 132,699 families) and full cousins (1,466,630 individuals from 597,317 families).

### Measures

#### Exposure

Several studies have used Rutter´s index of psychosocial adversity or its adapted version to assess the association with ADHD [8–10]. We used information from seven specific psychosocial adversities to create an index of cumulative psychosocial adversity in the family during the child’s first year of life. This index uses the available register data to approximate Rutter´s index [9], and has been used in previous studies as well. We considered the following dichotomous psychosocial adversity factors:

Parental bereavement, defined as any loss of first-degree relatives of the child´s parents (parents, partner, child, siblings) during the child’s first year of life, based on the Total Population Register.

Parental divorce during the child’s first year of life, defined as “yes” if the family situation has changed based on information from LISA the same year.

Parental financial problems during the child’s first year of life, defined as “yes” if the family had low economic status, based on information from LISA the same year. Low economic standard was defined as a household disposable income of less than 60% of the median income in the whole population in that particular year [31].

Parental low education during the child´s first year of life, defined as “yes” if at least one parent’s highest achieved educational level was less than 9 years of education, based on information from LISA the same year.

Any parental psychiatric history during child’s first year of life, based on ICD codes of parental neurodevelopmental and psychiatric conditions (Supplementary Table 1) identified from NPR the same year, versus none.

Any parental conviction for violent crime (e.g., homicide, assault, robbery, threats and violence against an officer, gross violation of a person’s integrity, unlawful coercion, unlawful threats, kidnaping, illegal confinement, arson, and intimidation) during the child´s first year of life, versus none. Data was obtained through the national Crime Register the same year.

Large family size, defined as 4 or more children in the family at the time of birth of the index person, based on information from the Medical Birth and Multi-Generation Registers.

All above mentioned psychosocial adversity factors were summarized into a cumulative psychosocial adversity index that was used as exposure, varying from 0 to 7. In the analysis, we treated this index as both a continuous and categorical variable, thus both assuming a linear dose-response pattern and allowing for deviations from linearity. Since few individuals were exposed to 4 or more factors (1293 exposed to 4 factors; 18 exposed to 5 factors; and no one was exposed to more than 5 factors) we merged these categories, resulting in index categories of 0, 1, 2, 3 and 4 or more factors for the categorical analysis.

#### Outcome

The outcome variables were defined as diagnosis of ADHD or autism after the age of 3 based on ICD-9 and ICD-10 in the NPR (Supplementary Table 2). Moreover, individuals with ADHD were also identified through PDR by Anatomical Therapeutic Chemical (ATC) (Supplementary Table 2) codes for ADHD medication. Both ADHD and autism were analyzed as time-to-event outcomes.

#### Covariates

We included parental age at child’s birth, child’s year of birth, and parent’s country of origin (Nordic, European, non-European) as covariates and adjusted for them in the analyses.

### Statistical analysis

The associations between cumulative psychosocial adversity and ADHD and autism were assessed with hazard ratios (HRs) and 95% confidence intervals (CI), using Cox regression. The children were followed from age 3 (baseline) until migration, death, end of follow up (December 31, 2013) or a diagnosis of ADHD/autism, whichever occurred first. Separate analyses were conducted for ADHD and autism, and for psychosocial adversity as continuous and categorical variables. We used Nelson–Aalen curves to visualize the probability of the outcome at a time interval between different levels of the cumulative exposure. First, the analyses for crude and adjusted models were conducted at a population level. Cluster robust standard errors were used in these analyses to account for dependence between individuals in the same family in the whole population. To adjust for unmeasured familial confounding, we repeated the analysis in different groups of relatives: full siblings who share 50% of their genes and all of their shared environment, half-siblings with 25% of shared genes and partly environment and full cousins with 12.5% shared genes and some of their environment [18]. In these analyses we used stratified Cox regression, where each group of relatives (e.g., each set of siblings) was entered as a separate stratum into the model grouped by the family identification code. Conceptually, the stratified Cox regression compares the risk of the outcome for individuals with different exposure levels within (i.e., stratifying on) the same group of relatives. By virtue of the stratification of groups of relatives, all factors that are constant within these groups (e.g., parental genetic make-up for full siblings) are automatically adjusted for. In the supplementary material (Supplementary Table 3), we provide the number of strata for which there is variation in the exposure and at least one child developed the outcome; most of the information about within-stratum associations is derived from these strata.

### Sensitivity analyses

To investigate if the effect of exposure to psychosocial adversity on ADHD was modified by autism, we fitted a model where ADHD was the outcome and autism was treated as a time-varying covariate, by including an interaction term between autism and the exposure in the model. Conversely, to investigate if the effect of exposure to psychosocial adversity on autism was modified by ADHD, we fitted a model where autism was the outcome and ADHD was treated as a time-varying covariate and included an interaction term in this model between ADHD and the exposure. In both these models we only used the continuous measure of psychosocial adversity.

To account for diagnostic uncertainty, we reran the analyses after having redefined the outcome: only those individuals diagnosed with ADHD or autism at least twice were considered as diagnosed, while individuals with one diagnosis were considered as undiagnosed. We also performed sex-stratified Cox regression on the general population to assess differences between boys and girls. Furthermore, in order to repeat the analysis among individuals with complete follow up from 2001, when information from both outpatient and inpatient care was available, we restricted the analysis to individuals turning 3 in 2001 or later (i.e., those who were born 1998 and onwards). Data management was performed in SAS software (Version 9.4 of the SAS System for Windows. Copyright © [2021] SAS Institute Inc). Analysis was performed in Stata (StataCorp. 2019. Stata Statistical Software: Release 16. College Station, TX: StataCorp LLC.). This study was approved by Stockholm’s Regional Ethical Review Board (Dnr 2013/862–31/5). Code availability: see supplementary materials.

## Results

**Fig. 1:**
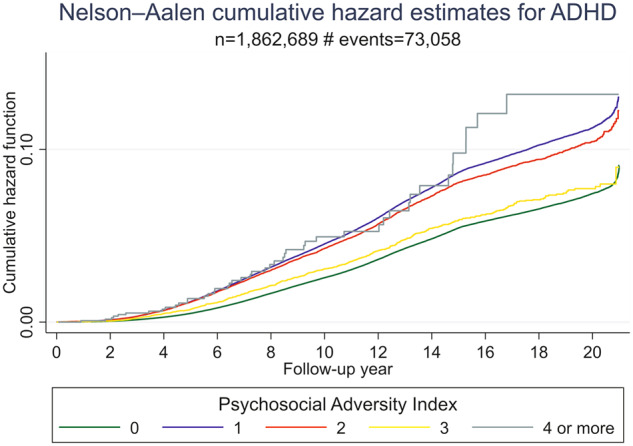
The cumulative psychosocial adversity index ranges from 0 to 4 or more factors. X-axis: Follow-up time from the age of 3 until the diagnosis of ADHD, death, migration, or 31 December 2009. Y-axis: Cumulative hazard function - the total accumulated risk of being diagnosed with ADHD over the follow-up time.

**Fig. 2:**
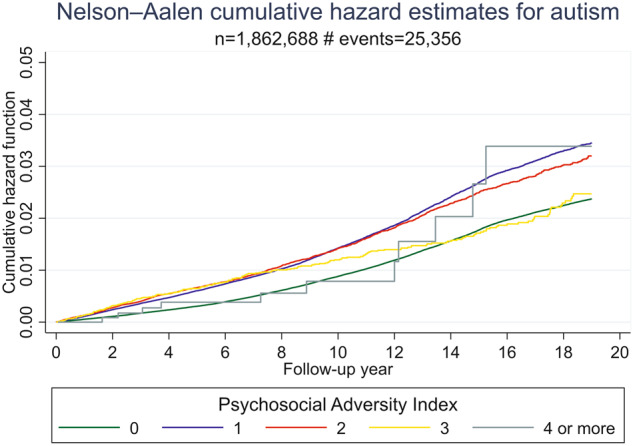
The cumulative psychosocial adversity index ranges from 0 to 4 or more factors. X-axis: Follow-up time from the age of 3 until the diagnosis of autism, death, migration, or 31 December 2009. Y-axis: Cumulative hazard function - the total accumulated risk of being diagnosed with autismover the follow-up time.

Descriptive characteristics of the study population are presented in Table 1. Median follow up time was 13.8 (interquartile range, IQR: 8.38; 19.40) years. During this time 73,058 participants (3.89%) were diagnosed with ADHD (median follow up time = 12.18 years (IQR: 9.09, 15.61)) and 25,356 with autism (1.35%) median follow up time = 13.8 years (IQR: 8.46, 19.55). The severity of exposure distributed as follows: 62.4% were unexposed, 36% were mildly exposed and only 1.6% experienced 4 or more exposure factors during the first year of life and did not differ between boys and girls (Table 1). Figs. 1 and 2 represent Nelson–Aalen cumulative hazard estimates of ADHD and autism diagnoses given different levels of exposure to cumulative psychosocial adversity.

**Table 1:** Descriptive information of study participants.

	Exposure to psychosocial adversity		
	No exposure	Mild exposure (1–3)	Severe exposure (≥4)
	*n* = 1,213,069	*n* = 663,521	*n* = 1311
Sex			
Male	621,313 (51.2%)	339,453 (51.2%)	665 (50.7%)
Female	591,756 (48.8%)	324,068 (48.8%)	646 (49.3%)
Birth year			
1990–1995	419,749 (34.6%)	127,366 (19.2%)	137 (10.5%)
1995–2000	299,922 (24.7%)	118,028 (17.8%)	220 (16.8%)
2000–2005	287,133 (23.7%)	143,830 (21.7%)	400 (30.5%)
2005–2010	206,265 (17.0%)	274,297 (41.3%)	554 (42.3%)
Length of follow up			
<7 years	149,169 (12.3%)	182,554 (27.5%)	427 (32.6%)
7–15 years	434,357 (35.8%)	276,382 (41.7%)	626 (47.7%)
>15 years	629,543 (51.9%)	204,585 (30.8%)	258 (19.7%)
Mean (SD), years	15.00 (0.01)	11.71 (0.01)	10.35 (0.14)
Median (IQR), years	15.44 (10.44; 20.25)	9.6569 (6.68; 16.79)	9.2 (6.17; 13.54)
Reasons for end of follow up			
Death			
No	1,210,428 (99.8%)	662,270 (99.8%)	1307 (99.7%)
Yes	2641 (0.2%)	1251 (0.2%)	4 (0.3%)
Migration			
No	1,174,447 (96.8%)	634,759 (95.7%)	1171 (89.3%)
Yes	38,622 (3.2%)	28,762 (4.3%)	140 (10.7%)
End of the study			
No	95,187 (7.8%)	60,426 (9.1%)	196 (15.0%)
Yes	1,117,882 (92.2%)	603,095 (90.9%)	1115 (85.0%)
ADHD diagnosis			
No	1,166,302 (96.1%)	637,276 (96.0%)	1265 (96.5%)
Yes	46,767 (3.9%)	26,245 (4.0%)	46 (3.5%)
Autism diagnosis			
No	1,196,861 (98.6%)	654,385 (98.6%)	1299 (99.1%)
Yes	16,208 (1.4%)	9136 (1.4%)	12 (0.9%)
Mother´s age at child’s birth, mean (SD)	29.2 (4.8)	29.5 (5.7)	33.3 (5.0)
Father´s age at child’s birth, mean (SD)	32.1 (5.5)	33.7 (7.0)	38.9 (6.6)
Mother’s country of birth			
Nordic	1,126,656 (92.9%)	474,532 (71.5%)	206 (15.7%)
European	36,396 (3.0%)	62,293 (9.4%)	264 (20.1%)
Non-European	49,988 (4.1%)	126,629 (19.1%)	841 (64.1%)
Missing	29 (0.0%)	67 (0.0%)	0 (0.0%)
Father’s country of birth			
Nordic	1,124,347 (92.7%)	461,520 (69.6%)	196 (15.0%)
European	40,760 (3.4%)	69,371 (10.5%)	271 (20.7%)
Non-European	47,857 (3.9%)	132,492 (20.0%)	844 (64.4%)
Missing	105 (0.0%)	138 (0.0%)	0 (0.0%)
Large family size			
No	1,213,069 (100.0%)	589,864 (88.9%)	99 (7.6%)
Yes	0 (0.0%)	73,657 (11.1%)	1212 (92.4%)
Bereavement		589,864 (88.9%)	99 (7.6%)
No	1,213,069 (100.0%)	656,682 (99.0%)	1258 (96.0%)
Yes	0 (0.0%)	6839 (1.0%)	53 (4.0%)
Crime conviction			
No	1,213,069 (100.0%)	663,049 (99.9%)	1279 (97.6%)
Yes	0 (0.0%)	472 (0.1%)	32 (2.4%)
Psychiatric history			
No	1,213,069 (100.0%)	652,607 (98.4%)	1078 (82.2%)
Yes	0 (0.0%)	10,914 (1.6%)	233 (17.8%)
Divorce			
No	1,203,170 (99.2%)	547,191 (82.5%)	145 (11.1%)
Yes	0 (0.0%)	111,290 (16.8%)	1165 (88.9%)
Missing	9899 (0.8%)	5040 (0.8%)	1 (0.1%)
Financial problems			
No	1,203,170 (99.2%)	547,191 (82.5%)	145 (11.1%)
Yes	0 (0.0%)	111,290 (16.8%)	1165 (88.9%)
Missing	9899 (0.8%)	5040 (0.8%)	1 (0.1%)
Low education			
No	1,189,765 (98.1%)	553,314 (83.4%)	45 (3.4%)
Yes	0 (0.0%)	73,887 (11.1%)	1262 (96.3%)
Missing	23,304 (1.9%)	36,320 (5.5%)	4 (0.3%)

*ADHD* attention-deficit hyperactivity disorder, *SD* standard deviation, *IQR* interquartile range.

### ADHD

In the general population, the cumulative psychosocial adversity index was associated with ADHD in a dose-response relationship, with adjusted HRs for ADHD ranging from 1.55 to 2.65 (Table 2). When the index was treated as a continuous variable, the HRs for ADHD increased 41% with each unit increase in the index. When the analyses were repeated in different groups of relatives, the HRs of ADHD attenuated towards the null with increasing levels of familial relationship (Table 2).

**Table 2:** Adjusted* hazard ratios (HR) for developing ADHD in general population and groups of relatives given different levels of exposure to cumulative psychosocial adversity.

	General population (1,877,901 observations, 73,058 cases)	Full cousins (2,184,849 observations, 85,436 cases)	Half-siblings (512,050 observations, 42,794 cases)	Full siblings (1,271,883 observations, 46,040 cases)
Psychosocial Adversity Index	Adjusted HR (95% CI)	Adjusted HR (95% CI)	Adjusted HR (95% CI)	Adjusted HR (95% CI)
1	1.55 (1.53–1.58)	1.18 (1.15–1.21)	1.04 (0.98–1.10)	1.01 (0.96–1.06)
2	1.88 (1.82–1.95)	1.33 (1.26–1.39)	1.04 (0.94–1.16)	1.08 (0.99–1.19)
3	1.89 (1.73–2.06)	1.29 (1.12–1.48)	0.98 (0.74–1.28)	1.16 (0.93–1.44)
≥4	2.65 (1.98–3.54)	1.99 (1.12–3.47)	0.60 (0.22–1.58)	1.56 (0.82–2.95)
Continuous	1.1 (1.39–1.42)	1.16 (1.13–1.18)	1.02 (0.98–1.07)	1.03 (0.99–1.07)

*Adjusted for parental age at child’s birth, child’s year of birth, and parental country of origin.

### Autism

The association between cumulative psychosocial adversity and autism did not follow any dose-response trend at a general population level. Adjusted HRs varied between 1.19 and 1.37 for exposure levels 1–3, adjusted HRs for the most exposed group was 1.04 which should be interpreted with caution due to lack of power (in the highly exposed group only 12 individuals got diagnosis with autism) (Table 3). Among relatives, a similar trend as seen in ADHD was observed for autism with estimates attenuated towards 1.

**Table 3:** Adjusted* hazard ratios for developing autism in general population and groups of relatives given different levels of exposure to cumulative psychosocial adversity.

	General population (1,877,901 observations, 25,356 cases)	Full cousins (2,184,849 observations, 28,572 cases)	Half-siblings (512,050 observations, 11,096 cases)	Full siblings (1,271,883 observations, 16,265 cases)
Psychosocial Adversity Index	Adjusted HR (95% CI)	Adjusted HR (95% CI)	Adjusted HR (95% CI)	Adjusted HR (95% CI)
1	1.33 (1.29–1.37)	1.07 (1.03–1.12)	1.06 (0.95–1.18)	1.01 (0.93–1.09)
2	1.37 (1.30–1.45)	1.15 (1.05–1.25)	1.01 (0.84–1.23)	1.14 (0.99–1.31)
3	1.19 (1.03–1.36)	1.16 (0.91–1.48)	0.84 (0.50–1.40)	0.99 (0.72–1.35)
≥4	1.04 (.59–1.84)	0.97 (0.32–2.95)	0.09 (0.01–0.85)	1.27 (0.53–3.04)
Continuous	1.20 (1.17–1.22)	1.07 (1.04–1.10)	1 (0.92–1.08)	1.04 (0.98–1.10)

*Adjusted for parental age at child’s birth, child’s year of birth, and parental country of origin

### Sensitivity analysis

The association between cumulative psychosocial adversity and ADHD was modified by autism (*p*-value_interaction term_ < .001)_._ The interaction analysis showed that cumulative psychosocial adversity increases the risk of ADHD regardless of autism. However, the effect was stronger in individuals with ADHD only (adjusted HR: 1.41, 95% CI: 1.40–1.43), compared to those with co-occurring ADHD and autism (adjusted HR: 1.15, 95% CI: 1.08–1.22) (Supplementary Table 4). The analysis further showed that cumulative psychosocial adversity increased the risk of autism among those without ADHD (adjusted HR: 1.33, 95% CI: 1.26–1.39) (Supplementary Table 5). However, there was no such association between cumulative psychosocial adversity and autism for those with co-occurring ADHD (adjusted HR: 0.97, 95% CI: 0.93–1.01) (Supplementary Table 5). HRs of getting the diagnosis of ADHD or autism were similar among those who have received the diagnosis after 2001 compared to those who were followed the whole time period (Supplementary Table 6).

The sensitivity analysis that aimed to account for the diagnostic uncertainty included only those who received at least 2 diagnoses of ADHD/autism showed no difference for ADHD and autism compared to the main analysis (Supplementary Table 7).

Separate analysis with stratification by sex showed that boys and girls had similar risks of developing ADHD with the dose-dependent effect related to experiencing cumulative psychosocial adversity (Supplementary Table 8). Sex-stratified analysis for autism showed that girls had a higher risk of being diagnosed compared to boys (Supplementary Table 9).

## Discussion

The main objective of this population-based study was to identify whether cumulative exposure to psychosocial adversity early in life increases the risk of ADHD and autism in children. Our findings on the general population level are in line with several previous studies showing that exposure to psychosocial adversities and traumatic events elevate the risk of ADHD and autism [10, 13, 32, 33]. We found a dose-dependent effect for ADHD but not for autism.

Importantly, for both outcomes, the estimates were attenuated towards the null with increasing level of familial relatedness, indicating that the associations were likely influenced by unmeasured familial confounding. Moreover, cumulative exposure to psychosocial adversity increased the risk of ADHD regardless of comorbidity with autism, but with a stronger effect among those without autism. For autism, the risk increase was observed only among individuals without ADHD.

In contrast with our findings at the population level, the most recent meta-analysis [25] emphasizes that almost no statistically significant results were found for early life exposure to pregnancy-related factors and parental characteristics and autism, while there were associations between low family income or transient income decline and externalizing behaviors and ADHD, although that study mostly inspected pregnancy-related factors. However, none of the previous studies have taken the co-occurrence between these conditions into account, and just a few of them were able to account for unmeasured genetic or environmental confounding. Given the high heritability of both ADHD and autism [7, 34], it is important to consider unmeasured confounding. Previous studies have shown that, in particular for individuals with ADHD, due to their genetic predisposition they tend to experience more adverse life events over the life span [35, 36]. A Norwegian study found that individuals with a higher genetic risk for ADHD are at increased risk for adverse life events [36]. In order to address this possibility, we compared different groups of relatives to account for unmeasured confounding shared between family members. This design accounts for unmeasured confounding to different degrees, e.g., comparing full siblings accounts for unmeasured confounding to a larger extent than cousins, since they have more environmental and genetic factors in common. In line with this, our findings showed that the associations between cumulative psychosocial adversity and ADHD and autism attenuated with increasing level of familial relatedness, indicating that the associations at a general population were likely influenced by unmeasured familial confounding. Future studies should be performed taking familial confounding into consideration.

This study has several strengths. It is population-based, and includes all children born in Sweden over a 20-year period. We used register-based data of high quality, with coverage of 85%–100% depending on the register [27, 28]. The outcome information is retrieved from a register that covers almost all in-patient care records since 1987 and out-patient records since 2001 which would account for half of the follow up time [28]. This is the largest study on cumulative psychosocial adversity and ADHD and autism to date, and the identification of relatives of different levels of kinship allowed us to adjust for unmeasured genetic and shared environmental confounding. Given that our measures of ADHD [37] and autism [38] were based on clinical diagnoses from specialist care, the reliability of the data is quite high, even though not all cases are reported in the register [38].

There are also several limitations to consider. Information on some of the psychosocial adversity factors included in the study, such as parental income and education, is based on register data that is updated once a year, which makes the timing of exposure somewhat imprecise. This might have influenced some sibling pairs where children were born close in time, resulting in siblings being concordant on exposure and therefore not contributing substantially to the analysis. The information about family situation only reflects those who separated after being married. We therefore had no information on separation of cohabiting partners, which is a common form of relationship in Sweden. Register-based data does not allow us to distinguish between symptomology and quantitative traits. Neither does it allow to explore the level of perception of experienced psychosocial adversity; therefore, we were not able to disentangle whether some factor had more impact, and these factors were equally weighted. Further studies with more detailed information on both exposure and outcome, including e.g., severity of symptoms, are clearly needed. The analyses on autism were hampered by the low number of children exposed to 4 or more factors, who also had autism. Therefore, we interpreted these results with caution, and in view of the overall pattern of results. Information on diagnoses in outpatient care was only available from 2001 and onwards. Since we only considered diagnoses after age 3, only individuals born 1999 or later have complete coverage in the outpatient register, which might lead to a misclassification of the outcome among those who were born before 1999. However, when we restricted the analyses to those who were diagnosed after 2001 the results were similar to the main analysis. There is also a possibility of carryover effects, where a younger sibling might be more likely to get diagnosed earlier if their older sibling has received a diagnosis of ADHD or autism [18]. However, we found commensurate results in cousins where such an effect should not be prominent.

### Clinical significance

This study may encourage clinicians and especially general practitioners to pay more attention to families that experience psychosocial adversity. If the association between psychosocial adversity and neurodevelopmental conditions is explained by familial confounding, the focus should be kept on early detection and intervention. This information can be used by policymakers and clinicians on vulnerable groups of children in need of more careful pediatric surveillance and screening for ADHD and autism. Early detection enables timely interventions, with the aim to prevent more severe psychiatric problems for children with ADHD and autism. The importance of familial confounding in this study highlights the need of further research into etiology of ADHD and autism to differentiate between the types of familial confounding, including genetic and shared environment.

## Conclusion

This is the first population-based study investigating the association between cumulative psychosocial adversity and both ADHD and autism, while accounting for unmeasured familial confounding. Our findings show that being exposed to cumulative psychosocial adversity in the family during the first year of life is associated with a greater risk of developing ADHD and autism. Associations were weaker among related individuals, indicating that they were at least partly explained by unmeasured familial confounding. The findings highlight the importance of accounting for unmeasured genetic and shared environmental confounding when studying the effect of psychosocial adversity on neurodevelopmental conditions [39, 40]. We advocate for using family-based designs, or other genetically informative designs, to account for unmeasured familial confounding when conducting studies on the etiology of ADHD and autism.

### Supplementary information

Supplementary materials
