# Correction: Association between cumulative psychosocial adversity in the family and ADHD and autism: a family-based cohort study

**DOI:** 10.1038/s41398-023-02595-z

**Published:** 2023-09-21

**Authors:** Aleksandra Kanina, Henrik Larsson, Arvid Sjölander, Agnieszka Butwicka, Mark J. Taylor, Miriam I. Martini, Paul Lichtenstein, Frida E. Lundberg, Brian M. D’ Onofrio, Mina A. Rosenqvist

**Affiliations:** 1 Department of Medical Epidemiology & Biostatistics, Karolinska Institutet, Stockholm, Sweden; 2 School of medical sciences, Örebro University, Örebro, Sweden; 3 Institute of Clinical Medicine, University of Oslo, Oslo, Norway; 4 Division of Mental Health Services, Akershus University Hospital, Lørenskog, Norway; 5 Department of Biostatistics and Translational Medicine, Medical University of Lodz, Lodz, Poland; 6 Department of Psychological and Brain Sciences, Indiana University, Bloomington, IN USA

**Keywords:** ADHD, Autism spectrum disorders

Correction to: *Translational Psychiatry* 10.1038/s41398-023-02571-7, published online 14 August 2023

The funding sources were given incomplete, the omitted funding sources are as follows:

Dr. Rosenqvist is supported by the Jeansons Foundation (Grant: JS2018-0006).

Dr. Rosenqvist is also supported by the Swedish Research Council (Grant: 2018-02119).

The original article has been corrected.

