# Supplementary material for: Association between cumulative psychosocial adversity in the family and ADHD and autism: a family-based cohort study
Source: Transl Psychiatry. 2023 Aug 14;13:282. doi: 10.1038/s41398-023-02571-7 (PMC10425335; doi:10.1038/s41398-023-02571-7)
Supplement: Supplementary file 1 — Supplementary materials [file 41398_2023_2571_MOESM1_ESM.docx]

**Association between cumulative psychosocial adversity in the family and ADHD and autism: a family-based cohort study.**

Aleksandra Kanina^1^, Henrik Larsson^1,2^, Arvid Sjölander^1^, Agnieszka Butwicka^1,3,4,5^, Mark J. Taylor^1^, Miriam I. Martini^1^, Paul Lichtenstein^1^, Frida E. Lundberg^1^, Brian M. D’Onofrio^1,6^, Mina A. Rosenqvist^1^

1.Department of Medical Epidemiology & Biostatistics, Karolinska Institutet, Stockholm, Sweden

2.School of medical sciences, Örebro University, Örebro, Sweden

3.Child and Adolescent Psychiatry Stockholm, Stockholm Health Care Services, Region Stockholm, Sweden

4.Department of Child Psychiatry, Medical University of Warsaw, Warsaw, Poland

5.Department of Biostatistics and Translational Medicine, Medical University of Lodz, Lodz, Poland

6.Department of Psychological and Brain Sciences, Indiana University, Bloomington, IN, USA

**Supplementary materials**

**Suppl. table 1**. ICD codes of parental psychiatric outcomes, identified from NPR.

| **Psychiatric outcome** | **ICD-8** | **ICD-9** | **ICD-10** |
| --- | --- | --- | --- |
|  | **(1969-1986)** | (**1987-1996)** | **(1997-)** |
| **Neurodevelopmental disorders** | | | |
| ADHD | - | 314 | F90 |
| Autism | - | 299 | F84 |
| Intellectual disability | 310-315 | 317-319 | F70-F79 |
| **Psychiatric conditions** | | | |
| Psychotic disorders | 295, 297-299; | 295, 297, 298; | F20-F29 |
| Mood disorders | 296, 300.40; | 296, 300E, 311 | F30-F39 |
| Anxiety, stress-related and somatoform disorders | 300.00-300.30,  300.50-300.99, 307; | 300, 300A-300D, 300F-300X, 308-309 | F40-F45, F48 |
| Eating disorders | - | 307B, 307F; | F50 |
| Substance misuse | 291, 303, 304; | 291, 303, 304, 305A, 305X | F10-F19 |
| Personality disorders | 301 | 301 | F60-F62, F69 |

**Suppl. table 2.** Inclusion and exclusion criteria for ADHD and autism diagnoses.

|  | **ADHD** | **Autism** |
| --- | --- | --- |
| **Inclusion criteria** | ICD-9: 314. ICD-10: F90.  ACT: Methylphenidate [N06BA04], Amphetamine [N06BA01], Dexamphetamine [N06BA02], Atomoxetine [N06BA09], Lisdexamfetamine [N06BA12] | ICD-9: 299A. ICD-10: F84.0, F84.1, F84.5, F84.8, F84.9 |
| **Exclusion criteria** |  | F84.2 Rett syndrome, F84.3 Dementia infantilis, Disintegrative psychosis, Heller's syndrome, Symbiotic psychosis, F84.4 Overactive disorder associated with mental retardation and stereotyped movements |

**Suppl. table 3.** Descriptive information on discordancy within groups of relatives who substantially contributed to analysis.

| Outcome | Number of informative* family strata in each group of relatives | | |
| --- | --- | --- | --- |
|  | Siblings | Half-siblings | Cousins |
| ADHD | 14485 | 164665 | 45294 |
| Autism | 5418 | 4752 | 16427 |

* Family strata with variation of exposure within the family, where at least one child received the outcome

**Suppl. table 4.** Adjusted hazard ratios (HR) for developing ADHD in children with and without autism given exposure to cumulative psychosocial adversity as a continuous measure.

|  | **HR (95% CI)** | **p-value** |
| --- | --- | --- |
| **Children without autism** (n=60,727) | 1.41 (1.40 – 1.43) | <0.001 |
| **Children with autism** (n=12,331) | 1.15 (1.08 – 1.22) | <0.001 |
| Models adjusted for parental age at child’s birth, child’s year of birth, and parental country of origin. p-value for interaction between exposure and autism <0.001. | | |

**Suppl. table 5.** Adjusted hazard ratios (HR) for developing autism in children with and without ADHD given exposure to cumulative psychosocial adversity as a continuous measure.

|  | **HR (95% CI)** | **p-value** |
| --- | --- | --- |
| **Children without ADHD** (n= 13,025) | 1.33 (1.26 – 1.39) | <0.001 |
| **Children with ADHD** (n=12,331) | 0.97 (0.93 – 1.01) | 0.196 |
| Models adjusted for parental age at child’s birth, child’s year of birth, and parental country of origin. p-value for interaction between exposure and ADHD <0.001. | | |

**Suppl. Table 6.** Crude and adjusted* hazard ratios for developing ADHD and autism after 2001 given different levels of exposure to cumulative psychosocial adversity.

|  | **Diagnosis of ADHD after 2001**  (n**=**33,066) | | **Diagnosis of autism after 2001**  **(**n=11,372) | |
| --- | --- | --- | --- | --- |
| **Psychosocial Adversity Index** | **Crude HR**  **(95% CI)** | **Adjusted HR**  **(95% CI)** | **Crude HR**  **(95% CI)** | **Adjusted HR**  **(95% CI)** |
| **1** | 1.66 (1.62 - 1.70) | 1.58 (1.54 - 1.62) | 1.51 (1.45 - 1.57) | 1.32 (1.26 - 1.38) |
| **2** | 1.58 (1.51 - 1.65) | 2.08 (1.00 - 2.18) | 1.61 (1.50 - 1.72) | 1.45 (1.34 - 1.56) |
| **3** | 1.16 (1.04 - 1.31) | 2.24 (1.98 – 2.53) | 1.48 (1.25 - 1.76) | 1.33 (1.11 - 1.60) |
| **≥4** | 1.44 (1.01 - 2.06) | 3.01 (2.09 – 4.33) | 0.77 (0.35 – 1.72) | 0.66 (0.29 – 1.47) |
| *Adjusted for parental age at child’s birth, child’s year of birth, and parental country of origin | | | | |

**Suppl. Table 7.** Crude and adjusted* hazard ratios for developing ADHD and autism for those who have received more than 1 diagnosis given different levels of exposure to cumulative psychosocial adversity.

|  | **Diagnosis of ADHD**  (n**=**58,270) | | **Diagnosis of autism**  (n**=**17,036) | |
| --- | --- | --- | --- | --- |
| **Psychosocial Adversity Index** | **Crude HR**  **(95% CI)** | **Adjusted HR**  **(95% CI)** | **Crude HR**  **(95% CI)** | **Adjusted HR**  **(95% CI)** |
| **1** | 1.62 (1.59 - 1.65) | 1.56 (1.53 - 1.59) | 1.48 (1.43 - 1.53) | 1.31 (1.26 - 1.36) |
| **2** | 1.48 (1.44 - 1.54) | 1.89 (1.83 - 1.96) | 1.45 (1.36 - 1.54) | 1.35 (1.26 - 1.44) |
| **3** | 1.06 (0.96 - 1.16) | 1.89 (1.72 - 2.09) | 1.14 (0.97 - 1.34) | 1.11 (0.94 - 1.32) |
| **≥4** | 1.81 (1.31 - 2.51) | 2.75 (1.98 - 3.82) | 0.94 (0.42 - 2.10) | 0.78 (0.35 - 1.74) |
| *Adjusted for parental age at child’s birth, child’s year of birth, and parental country of origin | | | | |

**Suppl. table 8.** Adjusted* hazard ratios for developing ADHD in boys and girls given different levels of exposure to cumulative psychosocial adversity.

|  | **Boys in general population**  (n=49,326) | | **Girls in general population**  (n**=**23,732) | |
| --- | --- | --- | --- | --- |
| **Psychosocial Adversity Index** | **Crude HR**  **(95% CI)** | **Adjusted HR**  **(95% CI)** | **Crude HR**  **(95% CI)** | **Adjusted HR**  **(95% CI)** |
| **1** | 1.63 (1.60 - 1.66) | 1.53 (1.50 - 1.56) | 1.67 (1.62 - 1.72) | 1.63 (1.58 - 1.68) |
| **2** | 1.54 (1.48 - 1.59) | 1.87 (1.80 – 1.95) | 1.52 (1.44 - 1.60) | 1.93 (1.82 - 2.04) |
| **3** | 1.14 (1.03 - 1.26) | 1.90 (1.72 - 2.11) | 1.06 (0.92 - 1.23) | 1.88 (1.61 - 2.19) |
| **≥4** | 1.58 (1.09 - 2.28) | 2.28 (1.58 - 3.31) | 2.58 (1.62 - 4.11) | 3.66 (2.29 - 5.84) |
| **Continuous** | 1.30 (1.29 - 1.32) | 1.40 (1.38 – 1.42) | 1.31 (1.29 – 1.33) | 1.44 (1.41 – 1.47) |
| Adjusted for parental age at child’s birth, child’s year of birth, and parental country of origin | | | | |

**Suppl. table 9.** Adjusted* hazard ratios for developing autism in boys and girls given different levels of exposure to cumulative psychosocial adversity.

|  | **Boys in general population**  (n=17,926) | | **Girls in general population**  (n=7,430) | |
| --- | --- | --- | --- | --- |
| **Psychosocial Adversity Index** | **Crude HR**  **(95% CI)** | **Adjusted HR**  **(95% CI)** | **Crude HR**  **(95% CI)** | **Adjusted HR**  **(95% CI)** |
| **1** | 1.57 (1.52 - 1.62) | 1.31 (1.26 - 1.36) | 1.49 (1.41 - 1.57) | 1.38 (1.31 - 1.46) |
| **2** | 1.50 (1.41 - 1.59) | 1.31 (1.23 - 1.40) | 1.46 (1.33 - 1.60) | 1.53 (1.38 - 1.69) |
| **3** | 1.17 (1.00 - 1.37) | 1.06 (0.90 - 1.25) | 1.26 (0.99 - 1.60) | 1.55 (1.21 - 1.99) |
| **≥4** | 1.17 (0.58 - 2.32) | 0.88 (0.44 - 1.75) | 1.62 (0.61 - 4.30) | 1.65 (0.62 - 4.39) |
| **Continuous** | 1.28 (1.26 – 1.30) | 1.17 (1.14 – 1.20) | 1.26 (1.22 – 1.30) | 1.27 (1.22 – 1.31) |
| *Adjusted for parental age at child’s birth, child’s year of birth, and parental country of origin | | | | |

Stata scripts are available through Open Science Framework (https://osf.io/r5v4h/?view_only=7afa2a8200b843a2b32574e7795d4406) for the data analysis using Cox regression on a simulated dataset of 100,000 individuals based on the original data by extracting this amount of observations and simulating the distribution of the variables to promote transparency and reproducibility of our results.
